# Effects of Bailing capsules combined with levothyroxine sodium on autoimmune thyroiditis

**DOI:** 10.1097/MD.0000000000040713

**Published:** 2024-11-29

**Authors:** Bai-Yu Su, Tao Wu, Li-Shuang Huo, Zhe Qu, Bu-Lang Gao

**Affiliations:** a Department of Endocrinology, Shijiazhuang People’s Hospital, Shijiazhuang, China; b Department of Neurosurgery, Shijiazhuang People’s Hospital, Shijiazhuang, China.

**Keywords:** adverse reaction, autoimmune thyroiditis, bailing capsules, levothyroxine sodium, treatment effect

## Abstract

To explore the clinical effects and adverse reactions of Bailing capsules combined with levothyroxine sodium for autoimmune thyroiditis, 70 patients with autoimmune thyroiditis were retrospectively enrolled and divided into the Bailing treatment group and the control, both consisting of 35 patients. The control group was treated with levothyroxine sodium alone, and the treatment group with Bailing capsules combined with levothyroxine sodium. The clinical efficacy, thyroid function indicators, antibodies and inflammatory indicators, and adverse drug reactions were analyzed. The total treatment effective rate was significantly (*P* = .04) higher in the treatment (94.29%) than in the control group (77.14%). After treatment, the levels of free triiodothyronine (8.69 ± 1.02 vs 6.70 ± 1.12 pmol/L) and free thyroxine (FT4) (20.05 ± 2.33 vs 13.00 ± 2.41 pmol/L) were significantly (*P* < .001) higher in the treatment group than those in the control group, the levels of thyroid peroxidase antibody (TPOAb) (298.70 ± 65.08 vs 735.15 ± 93.39 U/mL) and thyroglobulin antibodies (TgAb) (93.37 ± 21.10 vs 194.20 ± 37.48 U/mL) in the treatment group were significantly (*P* < .05) lower than those in the control group, and the interleukin-6 (IL-6) (82.83 ± 3.15 vs 97.17 ± 2.27 ng/L) and interleukin-17 (6.02 ± 0.67 vs 6.89 ± 0.72 ng/L) indicators in the treatment group were significantly (*P* < .05) lower than those in the control group. No significant (*P* = .393) difference was found in the adverse drug reaction. In conclusion, Bailing capsules combined with levothyroxine sodium are effective and safe for autoimmune thyroiditis, with good control of disease progression, improvement of thyroid function, reduction of thyroid function-related antibody level, and inhibition of inflammation.

## 1. Introduction

As a group of organ-specific autoimmune diseases mediated by T cells, autoimmune thyroiditis is at the early stage caused by increased levels of thyroid autoantibodies to damage the thyroid tissue.^[1–4]^ There are many factors to induce autoimmune thyroiditis, which are inseparable from heredity and environment.^[4–8]^ These patients may have hyperthyroidism, hypothyroidism, and other manifestations, including Hashimoto thyroiditis and toxic diffuse goiter. At present, there is no specific treatment for autoimmune thyroiditis, and medication is the main approach. Nonetheless, there is no consensus on the choice of drugs for the treatment. Levothyroxine sodium is one of the drugs of thyroid hormone and can maintain normal thyroid function of the human body. It is widely used in the replacement therapy of hypothyroidism. Its composition is consistent with thyroxine naturally secreted by the thyroid gland and can be converted into thyronine.^[9,10]^ Levothyroxine needs to be administered for a long time, and withdrawal of this medicine may result in a high risk of recurrence. Although levothyroxine can effectively regulate the concentration of thyroid hormones by binding with the receptor in the body, its immune regulation effect is limited and slow in achieving symptom relief. Moreover, levothyroxine monotherapy may not be effective in some patients in spite of having the thyroid stimulating hormone (TSH) being controlled within the reference range.^[11]^

Traditional Chinese medicine (TCM) has achieved some benefits in the treatment of many diseases because of the multiple active components to affect multiple organs and pathways.^[12–14]^ As a TCM drug, Bailing capsule (*Cordyceps sinensis*) is a new adjuvant drug with multiple functions of immune regulation and antitumor effects. It is a fermented cordyceps fungus powder preparation containing trace elements, amino acids, adenosine, and cordycepin. It can improve blood circulation, inhibit neurotransmitter release, and regulate adenylate cyclase activity. Currently, it has been used for the treatment of renal diseases.^[12,15,16]^ Bailing capsule has been shown to be able to adjust human immune function, prevent renal tubular atrophy, and repair renal tubular epithelial cells, thus improving the renal function.^[12,15–18]^ Bailing capsule can be combined with other Western medicines to increase the efficacy than the Western medicines alone in the treatment of renal diseases through decreasing urine protein and protecting renal function.^[15,19]^ Because of the immune function regulation, Bailing capsule may be effective in the regulation of the pathological mechanism of autoimmune thyroiditis. It was thus hypothesized that combined use of Bailing capsule and levothyroxine was able to effectively treat autoimmune thyroiditis compared with the levothyroxine sodium alone, and this study was conducted to investigate the effect of the combined use of Bailing capsule and levothyroxine sodium on autoimmune thyroiditis.

## 2. Materials and methods

This single-center retrospective study was approved by the ethics committee of Shijiazhuang People’s Hospital, and all patients had signed the informed consent to participate. Between January 2021 and April 2023, patients with autoimmune thyroiditis admitted to our hospital were enrolled and divided into the Bailing capsule treatment group and the control group. The inclusion criteria were patients with autoimmune thyroiditis, a movable clear-marginal hard neck mass, complete clinical data, age 18 to 65 years, but no relevant treatment before enrollment. The exclusion criteria were patients with other autoimmune diseases, abnormal liver and kidney function, mental illness, allergy to related drugs, and pregnancy or lactation.

In the control group, patients were treated with levothyroxine sodium alone using the levothyroxine sodium tablets (Shenzhen Zhonglian Pharmaceutical Co., Ltd. [Shenzhen, China], GYZZ H20010522, specification: 50 μg × 100 tablets) taken orally, once a day, and with an initial dose of 25 to 50 μg. Afterward, the dosage was adjusted according to the patients’ tolerance, with the treatment duration of 12 weeks. In the treatment group, Bailing capsule combined with levothyroxine sodium was used for the treatment. The treatment scheme of levothyroxine sodium was the same as that in the control group. Bailing capsule (Hangzhou Zhongmei Huadong Pharmaceutical Jiangdong Co., Ltd. [Hangzhou, China], ZYZZ Z10910036, specification: 0.5 g × 14 capsules) was administered orally, with 1 g each time, 3 times a day, and with the treatment duration of 12 weeks. During the treatment period, both groups of patients had a low-salt diet.

The treatment effect was divided into 3 levels: marked effect, moderate effect, and ineffectiveness. The marked effect was defined if the symptoms disappeared, and the thyroid antibody indicators decreased by 70% or more. For the moderate effect, the symptoms were alleviated, with the thyroid antibody indicators being decreased by 30% to 70%. In patients with ineffectiveness, the symptoms remained and the thyroid antibody indicators were decreased <30%. The total effective rate is equal to the number of patients with the marked and moderate effect divided by the total number of patients.

In the test of the thyroid function, the fasting venous blood of the 2 groups of patients was collected, and the serum was extracted after high-speed centrifugation. The automatic chemical reflex immune analyzer and matching reagents were used to measure the levels of TSH, free triiodothyronine (FT3), free thyroxine (FT4), TPOAb, and TgAb. After the serum was separated, the levels of IL-6 and interleukin-17 (IL-17) were measured by ELISA.

In the evaluation of the safety of the treatment, the adverse drug reaction was analyzed, and the incidence rate was used as the safety evaluation index, involving gastrointestinal discomfort, pharyngeal discomfort, and palpitations. Follow-up was performed for all patients at the clinics or with phone contact at an interval of 7 days once to monitor improvement of clinical symptoms, patients’ compliance with drug administration, and adjustment of drug dosage according to the symptoms.

### 2.1. Statistical analysis

The statistical analysis was conducted using the SPSS version 25.0 (IBM, Chicago, IL). Measurement data in the normal distribution were presented as mean ± standard deviation and tested with the *t* test. Categorical data were presented as frequency and percentage and tested with the Chi-square test. The significant *P* level was set at <.05.

## 3. Results

Seventy patients with autoimmune thyroiditis were enrolled and divided into the Bailing treatment group (n = 35) and the control group (n = 35). The treatment group consisted of 22 males and 13 females aged 33 to 65 (48.69 ± 4.35) years, with a disease course of 6 to 36 (18.71 ± 2.05) months. The control group was composed of 10 males and 25 females aged 35 to 64 (48.35 ± 4.10) years, with a disease course of 6 to 37 (18.65 ± 2.31) months. No significant (*P* > .05) differences were detected in the baseline data between the 2 groups.

In the control group, the marked effect was achieved in 14 (40%) patients, moderate effect in 13 (37.14%), and ineffectiveness in 8 (22.86%), whereas in the treatment group, the marked effect was achieved in 20 (57.14%) patients, moderate effect in 13 (37.14%), and ineffectiveness in 2 (5.71%) (Table 1). The total effective rate was significantly (*P* = .04) higher in the treatment group (94.29%) than in the control group (77.14%) (Table 1).

**Table 1:** Treatment effect in the 2 groups (n, %).

Groups	No.	Marked effect	Moderate effect	Ineffectiveness	Total effective rate
Control	35	14 (40.00)	13 (37.14)	8 (22.86)	27 (77.14)
Treatment	35	20 (57.14)	13 (37.14)	2 (5.71)	33 (94.29)
χ^2^					4.20
*P*					.04

There was no significant (*P* > .05) difference in the thyroid function indicators between the 2 groups before treatment. After treatment, the TSH levels in the treatment group (9.87 ± 0.95 mU/L) were lower than those in the control group (10.13 ± 0.71 mU/L), whereas the levels of FT3 and FT4 were significantly (*P* < .001) higher in the treatment group (8.69 ± 1.02 and 20.05 ± 2.33 pmol/L, respectively) than those in the control group (6.70 ± 1.12 and 13.00 ± 2.41 pmol/L, respectively) (Table 2).

**Table 2:** Thyroid function indicators in 2 groups (mean ± standard deviation).

Groups	No.	TSH (mU/L)		FT3 (pmol/L)		FT4 (pmol/L)	
		Before treatment	After treatment	Before treatment	After treatment	Before treatment	After treatment
Control	35	25.31 ± 2.67	10.13 ± 0.71	2.24 ± 0.76	6.70 ± 1.12	3.78 ± 1.19	13.00 ± 2.41
Treatment	35	25.26 ± 2.45	9.87 ± 0.95	2.26 ± 0.74	8.69 ± 1.02	3.92 ± 1.23	20.05 ± 2.33
*T*		0.0817	1.2970	0.1115	7.7717	0.4840	12.4422
*P*		.9352	.1990	.9115	<.001	.6300	<.001

FT3 = free triiodothyronine, FT4 = free thyroxine, TSH = thyroid stimulating hormone.

No significant (*P* > .05) difference was detected in the thyroid antibodies before treatment between the 2 groups, with the TPOAb and TgAb of 914.15 ± 134.71 and 254.96 ± 41.34 U/mL in the control group and 916.26 ± 131.59 and 257.05 ± 38.76 U/mL in the treatment group, respectively. After treatment, the levels of TPOAb and TgAb in the treatment group (298.70 ± 65.08 and 93.37 ± 21.10 U/mL, respectively) were significantly (*P* < .05) lower than those in the control group (735.15 ± 93.39 and 194.20 ± 37.48 U/mL, respectively) (Table 3).

**Table 3:** Thyroid antibodies (U/mL, mean ± standard deviation).

Groups	No.	TPOAb		TgAb	
		Before treatment	After treatment	Before treatment	After treatment
Control	35	914.15 ± 134.71	735.15 ± 93.39	254.96 ± 41.34	194.20 ± 37.48
Treatment	35	916.26 ± 131.59	298.70 ± 65.08	257.05 ± 38.76	93.37 ± 21.10
*t*		0.0663	22.6837	0.2182	13.8689
*P*		.9473	.0000	.8279	.0000

TgAb = thyroglobulin antibodies, TPOAb = thyroid peroxidase antibody.

No significant (*P* > .05) difference was found in the levels of inflammatory factors before treatment between the 2 groups, with the IL-6 and IL-17 of 65.47 ± 6.81 and 7.29 ± 1.05 ng/L in the control group and 65.16 ± 7.43 and 7.32 ± 1.10 ng/L in the treatment group, respectively. After treatment, the IL-6 and IL-17 in the treatment group (82.83 ± 3.15 and 6.02 ± 0.67 ng/L, respectively) were significantly (*P* < .05) lower than those in the control group (97.17 ± 2.27 and 6.89 ± 0.72 ng/L, respectively) (Table 4).

**Table 4:** Inflammatory factors in the 2 groups (ng/L, mean ± standard deviation).

Groups	No.	IL-6		IL-17	
		Before treatment	After treatment	Before treatment	After treatment
Control	35	65.47 ± 6.81	82.83 ± 3.15	7.29 ± 1.05	6.89 ± 0.72
Treatment	35	65.16 ± 7.43	97.17 ± 2.27	7.32 ± 1.10	6.02 ± 0.67
*t*		0.1820	26.12	0.1167	5.2333
*P*		.8562	<.0001	.9074	<.0001

IL-6 = interleukin 6, IL-17 = interleukin-17.

In safety evaluation, no significant (*P* = .393) difference was found in the adverse reaction (Table 5). In the control group, gastrointestinal discomfort occurred in 1 (2.86%) patient and palpitations took place in 1 (2.86%). In the treatment group, gastrointestinal discomfort happened in 2 (5.71%) patients and pharyngeal discomfort in 1 (2.86%).

**Table 5:** Adverse drug reactions (n, %).

Groups	No.	Gastrointestinal discomfort	Pharyngeal discomfort	Palpitations	Total incidence
Control	35	1 (2.86)	0 (3.33)	1 (2.86)	2 (5.71)
Treatment	35	2 (5.71)	1 (2.86)	0 (0.00)	3 (8.57)
*χ* ^2^					0.729
*P*					.393

## 4. Discussion

In this study exploring the clinical effects and safety of Bailing capsule combined with the levothyroxine sodium in the treatment of autoimmune thyroiditis compared with the levothyroxine sodium alone, it was found that Bailing capsule combined with the levothyroxine sodium was effective and safe for autoimmune thyroiditis, with good control of disease progression, improvement of thyroid function, reduction of thyroid antibody levels, and inhibition of inflammation.

Autoimmune thyroiditis is a disease characterized by abnormal immune function of the body and targets thyroid follicular epithelial cell antigen components, leading to tissue cell damage, inflammatory reaction, and finally thyroid dysfunction.^[1–4]^ This disease mostly occurs in women aged 30 to 50 years, and has the characteristics of slow onset, a long disease course, and possible symptoms of goiter. If not treated in time, it may progress to diffuse nodular changes in the thyroid, increasing the treatment difficulty and clinical damage. Previous studies have shown that heredity and environment are the key factors leading to the occurrence of autoimmune thyroiditis, for which no specific treatment methods exist.^[4–8]^ Drug therapy is the first choice, and in particular, hormone replacement therapy is often used in clinical practice.^[9,10]^ Nevertheless, there are still major disputes about its effect and long-term drug safety, and more feasible treatment plans still need to be explored.

The TCM treatment has unique advantages and plays an important role in the control of various diseases.^[12,13,15–25]^ The current development of traditional Chinese patent medicines and simple preparations makes TCM treatment more convenient and popular. Integrated TCM and Western medicine treatment can display multiple advantages, improve clinical efficacy, and have high application value and significance in autoimmune thyroiditis.

The TCM classifies autoimmune thyroiditis as in the “stone gall” category. It believes that the main causes of the disease are congenital endowment deficiency and kidney qi deficiency, and the acquired improper conditioning leads to the invasion of wind and evil. Therefore, the treatment should be based on the main principle of supplementing the essence and benefiting the qi, strengthening the body and its foundation.^[26]^ Bailing capsule is a kind of capsule preparation of traditional Chinese patent medicines composed of fermented *C sinensis* powder. It is a tonic, with the effects of tonifying the lungs and kidneys and replenishing vital energy. It can improve the immune function of the body,^[12,16,17,26–28]^ suitable for the treatment of autoimmune thyroiditis.

Based on the TCM theory, Bailing capsule was applied to the treatment of autoimmune thyroiditis in combination with levothyroxine sodium in our study. The results showed that the treatment efficiency of the treatment group was significantly higher than that of the control group. After treatment, the TSH levels in the treatment group were lower than those in the control group, whereas the FT3 and FT4 levels were significantly higher than those in the control group. The TPOAb and TgAb levels in the treatment group were significantly lower than those in the control group. It can be seen that the combination of the 2 drugs has a more significant therapeutic effect compared to the mono-levothyroxine sodium replacement therapy, and can improve the thyroid function and antibody expression of patients, beneficial for reducing the thyroid function damage. This is because the Bailing capsule and the levothyroxine sodium work from different mechanisms. They complement each other and will not have adverse effects on each other. The levothyroxine sodium regulates the level of thyroid hormones and can improve the therapeutic effect by reaching directly the lesion from the perspective of TCM. From the perspective of modern pharmacology, Bailing capsule is mainly composed of fermented *C sinensis* preparation, which can improve the respiratory capacity of mitochondria in cells, help restore damaged thyroid cells and physiological functions, play an immunomodulatory role, reduce the level of thyroid hormones, and stabilize the function of cell membrane, with significant advantages.^[29]^

The immunity of patients with autoimmune thyroiditis decreases. Bailing capsule can regulate their immune function and alleviate their cellular immune function disorder, and the combination of this drug with the levothyroxine sodium can coordinate and enhance the effect of both drugs. The lymphocytes of patients with this disease exhibit a large amount of thyroid antibodies due to functional disorders, which disrupt thyroid function and trigger inflammatory reactions.^[1–4]^ After treatment, the IL-6 and IL-17 indicators in the treatment group were significantly lower than those in the control group. It is suggested that Bailing capsule can not only regulate autoimmunity and improve thyroid function, but also inhibit inflammatory reactions, with the results of achieving better anti-inflammatory effects and alleviating disease symptoms. In addition, patients with autoimmune thyroiditis have poor immunity and limited body tolerance, so we should not blindly pursue efficacy while ignoring safety in the treatment process. The occurrence of adverse reactions not only causes physical and mental discomfort in patients, but also may affect the smooth progress of treatment, which is not helpful for an ideal prognosis. In our study, no significant difference was detected in the drug adverse reaction between the 2 groups, indicating that Bailing capsule can improve the efficacy, but will not increase the clinical damage or adverse effects. The drug is natural in origin and safe in composition. The combination with the levothyroxine sodium meets the safety needs of patients.

Some limitations existed in our study, including the retrospective and 1-center study design, a small cohort of patients, Chinese patients enrolled only, a short follow-up period, and no randomization of the patients to different groups, which may all affect the generalization of the outcomes. The retrospective design may introduce potential selection and recall bias. The small sample of this study may not provide sufficient statistical power for analysis, and together with the single-center study design (center-specific biases may be introduced in patient selection, treatment protocols, and outcomes), may limit the generalizability of the results. The lack of randomization in assigning patients to different groups may raise concerns about confounding factors affecting the outcomes. The short follow-up period may not adequately capture long-term effects or later-onset adverse reactions. Moreover, we just made a primary investigation on the mechanism of Bailing capsules for the treatment of autoimmune thyroiditis based on the changes in the thyroid function, antibodies, and interleukin 6 and 17, and detailed exploration of the Bailing capsule mechanism needs further studies in the future. Future randomized, controlled, multicenter studies involving multiple races and ethnicities in a large number with a long-time follow-up will have to be conducted to validate the findings, explore the detailed mechanism of treatment, obtain better outcomes, and enhance generalizability.

In conclusion, Bailing capsule combined with the levothyroxine sodium are effective and safe in the treatment of autoimmune thyroiditis, with good control of disease progression, improvement of thyroid function, reduction of thyroid function-related antibody levels, and inhibition of inflammation even though large controlled randomized clinical trials are necessary to confirm the outcomes.

## Author contributions

**Conceptualization:** Bai-Yu Su.

**Data curation:** Bai-Yu Su, Tao Wu, Li-Shuang Huo, Zhe Qu, Bu-Lang Gao.

**Formal analysis:** Bai-Yu Su, Tao Wu, Li-Shuang Huo, Zhe Qu, Bu-Lang Gao.

**Investigation:** Bai-Yu Su, Tao Wu, Li-Shuang Huo, Zhe Qu, Bu-Lang Gao.

**Methodology:** Bai-Yu Su, Tao Wu, Li-Shuang Huo.

**Software:** Bai-Yu Su, Tao Wu, Li-Shuang Huo, Bu-Lang Gao.

**Supervision:** Bai-Yu Su, Tao Wu, Zhe Qu, Bu-Lang Gao.

**Validation:** Bai-Yu Su, Tao Wu, Li-Shuang Huo, Zhe Qu, Bu-Lang Gao.

**Visualization:** Bai-Yu Su, Tao Wu, Li-Shuang Huo, Zhe Qu, Bu-Lang Gao.

**Writing—original draft:** Bai-Yu Su.

**Project administration:** Tao Wu, Zhe Qu.

**Resources:** Li-Shuang Huo, Zhe Qu, Bu-Lang Gao.

**Writing—review & editing:** Bu-Lang Gao.

## References

[1] Ajjan RA Weetman AP. The pathogenesis of Hashimoto’s thyroiditis: further developments in our understanding. Horm Metab Res. 2015;47:702–10. 26361257 10.1055/s-0035-1548832

[2] Amino N. Autoimmunity and hypothyroidism. Baillieres Clin Endocrinol Metab. 1988;2:591–617. 3066320 10.1016/s0950-351x(88)80055-7

[3] Bhakat B Pal J Das S. A prospective study to evaluate the possible role of cholecalciferol supplementation on autoimmunity in Hashimoto’s thyroiditis. J Assoc Physicians India. 2023;71:1.

[4] Ippolito S Di Dalmazi G Pani F Sabini E Caturegli P. Distinct cytokine signatures in thyroiditis induced by PD-1 or CTLA-4 blockade: insights from a new mouse model. Thyroid. 2021;31:1839–49. 34598661 10.1089/thy.2021.0165 PMC8721507

[5] Effraimidis G Tijssen JG Wiersinga WM. Discontinuation of smoking increases the risk for developing thyroid peroxidase antibodies and/or thyroglobulin antibodies: a prospective study. J Clin Endocrinol Metab. 2009;94:1324–8. 19141579 10.1210/jc.2008-1548

[6] Hadj-Kacem H Rebuffat S Mnif-Feki M Belguith-Maalej S Ayadi H Péraldi-Roux S. Autoimmune thyroid diseases: genetic susceptibility of thyroid-specific genes and thyroid autoantigens contributions. Int J Immunogenet. 2009;36:85–96. 19284442 10.1111/j.1744-313X.2009.00830.x

[7] Kolypetri P King J Larijani M Carayanniotis G. Genes and environment as predisposing factors in autoimmunity: acceleration of spontaneous thyroiditis by dietary iodide in NOD.H2(h4) mice. Int Rev Immunol. 2015;34:542–56. 26287317 10.3109/08830185.2015.1065828

[8] Weetman AP. An update on the pathogenesis of Hashimoto’s thyroiditis. J Endocrinol Invest. 2021;44:883–90. 33332019 10.1007/s40618-020-01477-1 PMC8049926

[9] Wilson SA Stem LA Bruehlman RD. Hypothyroidism: diagnosis and treatment. Am Fam Physician. 2021;103:605–13. 33983002

[10] Kahaly GJ Gottwald-Hostalek U. Use of levothyroxine in the management of hypothyroidism: a historical perspective. Front Endocrinol (Lausanne). 2022;13:1054983. 36407302 10.3389/fendo.2022.1054983 PMC9666762

[11] Razvi S Mrabeti S Luster M. Managing symptoms in hypothyroid patients on adequate levothyroxine: a narrative review. Endocr Connect. 2020;9:R241–50. 33112818 10.1530/EC-20-0205 PMC7774765

[12] Zhang Q Xiao X Li M Yu M Ping F. Bailing capsule (*Cordyceps sinensis*) ameliorates renal triglyceride accumulation through the PPARα pathway in diabetic rats. Front Pharmacol. 2022;13:915592. 36091833 10.3389/fphar.2022.915592 PMC9453879

[13] Dai B Wang ZZ Zhang H Han M-X Zhang G-X Chen J-W. Antihypertensive properties of a traditional Chinese medicine GAO-ZI-YAO in elderly spontaneous hypertensive rats. Biomed Pharmacother. 2020;131:110739. 32932045 10.1016/j.biopha.2020.110739

[14] Wu J Wei Z Cheng P. Rhein modulates host purine metabolism in intestine through gut microbiota and ameliorates experimental colitis. Theranostics. 2020;10:10665–79. 32929373 10.7150/thno.43528 PMC7482825

[15] Hu X Wang J Yang H. Bailing capsule combined with alpha-ketoacid tablets for stage 3 chronic kidney disease: protocol of a double-blinded, randomized, controlled trial. Medicine (Baltim). 2021;100:e25759. 10.1097/MD.0000000000025759 PMC8136994 34011035

[16] Xu J Yuan Q Wu K Li X Zhao Y Li X. Effects of Bailing capsule on diabetic nephropathy based on UPLC-MS urine metabolomics. RSC Adv. 2019;9:35969–75. 35540588 10.1039/c9ra05046a PMC9074918

[17] Sheng X Dong Y Cheng D Wang N Guo Y. Efficacy and safety of Bailing capsules in the treatment of type 2 diabetic nephropathy: a meta-analysis. Ann Palliat Med. 2020;9:3885–98. 33222468 10.21037/apm-20-1799

[18] Wu J Yan W Wu X Hong D Lu X Rao Y. Protective effects of Corbrin capsule against permanent cerebral ischemia in mice. Biomed Pharmacother. 2020;121:109646. 31739162 10.1016/j.biopha.2019.109646

[19] Yu W Duan S Yu Z. The effect of Bailing capsules combined with losartan to treat diabetic glomerulosclerosis and the combination’s effect on blood and urine biochemistry. Am J Transl Res. 2021;13:6873–80. 34306438 PMC8290721

[20] Wang ZQ Cui YH Huang Y. Herb-partitioned moxibustion regulated the miRNA expression profile in the thyroid tissues of rats with experimental autoimmune thyroiditi. J Tradit Chin Med. 2021;41:789–98. 34708638 10.19852/j.cnki.jtcm.2021.05.013

[21] Liu Z Song N Li M. Based on mRNA sequencing techniques to explore the molecular mechanism of Buzhong Yiqi decoction for autoimmune thyroiditis. Comb Chem High Throughput Screen. 2024;27:408–19. 37070455 10.2174/1386207326666230417120421

[22] Ma HD Deng YR Tian Z Lian Z-X. Traditional Chinese medicine and immune regulation. Clin Rev Allergy Immunol. 2013;44:229–41. 22826112 10.1007/s12016-012-8332-0

[23] Li J Li J Zhang F. The immunoregulatory effects of Chinese herbal medicine on the maturation and function of dendritic cells. J Ethnopharmacol. 2015;171:184–95. 26068430 10.1016/j.jep.2015.05.050

[24] Wang Y Zhang X Wang Y. Application of immune checkpoint targets in the anti-tumor novel drugs and traditional Chinese medicine development. Acta Pharm Sin B. 2021;11:2957–72. 34729298 10.1016/j.apsb.2021.03.004 PMC8546663

[25] Zhao M Liu L Liu F. Traditional Chinese medicine improves myasthenia gravis by regulating the symbiotic homeostasis of the intestinal microbiota and host. Front Microbiol. 2022;13:1082565. 36687653 10.3389/fmicb.2022.1082565 PMC9852828

[26] Nana Wang XL Deng W Huang H Chen X. The clinical effect of Bailing capsule and Prunella oral liquid combined with euthyrox in the treatment of Hashimoto’s hypothyroidism. J Hubei Univ Med. 2021;40:134–8.

[27] Wang W Zhang XN Yin H. Effects of Bailing capsules for renal transplant recipients: a retrospective clinical study. Chin Med J (Engl). 2013;126:1895–9. 23673106

[28] Xu H Li S. [Pharmacological effects of Bailing capsule and its application in lung disease research]. Zhongguo Zhong Yao Za Zhi. 2010;35:2777–81. 21246839

[29] Yefeng ZHD. Changes of three serum markers in patients with autoimmune thyroid disease before and after treatment with two drugs. Int J Med Laboratory. 2019;40:2002–4.

